# Updated Surveillance Metrics and History of the COVID-19 Pandemic (2020-2023) in Canada: Longitudinal Trend Analysis

**DOI:** 10.2196/53218

**Published:** 2024-12-05

**Authors:** Scott A Wu, Alan G Soetikno, Egon A Ozer, Sarah B Welch, Yingxuan Liu, Robert J Havey, Robert L Murphy, Claudia Hawkins, Maryann Mason, Lori A Post, Chad J Achenbach, Alexander L Lundberg

**Affiliations:** 1 Feinberg School of Medicine Northwestern University Chicago, IL United States; 2 Department of Medicine, Division of Infectious Diseases Feinberg School of Medicine Northwestern University Chicago, IL United States; 3 Center for Pathogen Genomics and Microbial Evolution Robert J Havey, MD Institute for Global Health Northwestern University Chicago, IL United States; 4 Buehler Center for Health Policy and Economics Robert J Havey, MD Institute for Global Health Northwestern University Chicago, IL United States; 5 Department of Emergency Medicine Feinberg School of Medicine Northwestern University Chicago, IL United States; 6 Robert J Havey, MD Institute for Global Health Northwestern University Chicago, IL United States; 7 Department of Medicine, General Internal Medicine and Geriatrics Feinberg School of Medicine Northwestern University Chicago, IL United States; 8 Center for Global Communicable and Emerging Infectious Diseases Robert J Havey, MD Institute for Global Health, Northwestern University Chicago, IL United States

**Keywords:** SARS-CoV-2, COVID-19, Canada, pandemic, surveillance, transmission, acceleration, deceleration, dynamic panel, generalized method of moments, GMM, Arellano-Bond, 7-day lag, k, metrics, epidemiology, dynamic, genomic, historical context, outbreak threshold

## Abstract

**Background:**

This study provides an update on the status of the COVID-19 pandemic in Canada, building upon our initial analysis conducted in 2020 by incorporating an additional 2 years of data.

**Objective:**

This study aims to (1) summarize the status of the pandemic in Canada when the World Health Organization (WHO) declared the end of the public health emergency for the COVID-19 pandemic on May 5, 2023; (2) use dynamic and genomic surveillance methods to describe the history of the pandemic in Canada and situate the window of the WHO declaration within the broader history; and (3) provide historical context for the course of the pandemic in Canada.

**Methods:**

This longitudinal study analyzed trends in traditional surveillance data and dynamic panel estimates for COVID-19 transmissions and deaths in Canada from June 2020 to May 2023. We also used sequenced SARS-CoV-2 variants from the Global Initiative on Sharing All Influenza Data (GISAID) to identify the appearance and duration of variants of concern. For these sequences, we used Nextclade nomenclature to collect clade designations and Pangolin nomenclature for lineage designations of SARS-CoV-2. We used 1-sided *t* tests of dynamic panel regression coefficients to measure the persistence of COVID-19 transmissions around the WHO declaration. Finally, we conducted a 1-sided *t* test for whether provincial and territorial weekly speed was greater than an outbreak threshold of 10. We ran the test iteratively with 6 months of data across the sample period.

**Results:**

Canada’s speed remained below the outbreak threshold for 8 months by the time of the WHO declaration ending the COVID-19 emergency of international concern. Acceleration and jerk were also low and stable. While the 1-day persistence coefficient remained statistically significant and positive (1.074; *P*<.001), the 7-day coefficient was negative and small in magnitude (–0.080; *P*=.02). Furthermore, shift parameters for either of the 2 most recent weeks around May 5, 2023, were negligible (0.003 and 0.018, respectively, with *P* values of .75 and .31), meaning the clustering effect of new COVID-19 cases had remained stable in the 2 weeks around the WHO declaration. From December 2021 onward, Omicron was the predominant variant of concern in sequenced viral samples. The rolling 1-sided *t* test of speed equal to 10 became entirely insignificant from mid-October 2022 onward.

**Conclusions:**

While COVID-19 continues to circulate in Canada, the rate of transmission remained well below the threshold of an outbreak for 8 months ahead of the WHO declaration. Both standard and enhanced surveillance metrics confirm that the pandemic had largely ended in Canada by the time of the WHO declaration. These results can inform future public health interventions and strategies in Canada, as well as contribute to the global understanding of the trajectory of the COVID-19 pandemic.

## Introduction

### Background

COVID-19, the disease caused by the virus SARS-CoV-2, was first identified in Wuhan, China, in the autumn of 2019 [1-3]. Canada’s first confirmed case of COVID-19 occurred on January 27, 2020, in Winnipeg [4]. Our research team conducted an initial analysis of the pandemic’s impact in Canada 1 year into its outbreak [5]. This study presents updated surveillance and statistical modeling spanning 2 additional years and is restricted to the pandemic in Canada.

At the start of the COVID-19 pandemic, Canada effectively controlled SARS-CoV-2 transmissions with a rapid policy response [5,6]. At the end of 2020, however, a wave of transmissions resulted in an outbreak. The early outbreak was limited in severity and duration compared with the outbreak in the United States, which shares an extensive border with Canada [7,8]. Differences in the trajectory of the pandemic across countries can reflect a range of factors, such as policy response and geography, and these trajectories can inform preparation for future outbreaks and pandemics. This study provides an updated analysis of the progression of COVID-19 in Canada approximately midway through 2023. On May 5, 2023, the World Health Organization (WHO) and Director-General Ghebreyesus declared the end of COVID-19 as a public health emergency of international concern following a recommendation from the COVID-19 Emergency Committee [9,10]. We aim to compare how the pandemic evolved in Canada before and after the declaration.

### Traditional Surveillance Versus Enhanced Surveillance

Field epidemiology defines terms of pandemic, epidemic, outbreak, and endemic based on transmission metrics and geographical distribution. While public health surveillance data suffer from incomplete case ascertainment, they provide the best proxy to monitor and track the spread of disease within a population in near real time, which is essential to timely responses to health threats [11].

Public health surveillance is defined as the “ongoing, systematic collection, analysis, and interpretation of health-related data essential for planning and evaluating public health practices” [12]. Surveillance not only reveals the impact of diseases or social conditions on public health but also generates research inquiries and provides guidance for further investigation [13-16]. It enables us to compare disease burdens across geographical regions and identifies the most affected areas. This impact assessment includes the measurement of disease contraction rates, mortality rates, and associated costs.

However, traditional surveillance has its limitations, which this study addresses. Traditional surveillance essentially provides a historical snapshot of past events [13-15], making it static and backward looking. During a rapidly evolving pandemic, policy makers and public health practitioners require insight into what lies ahead. They need to know if an outbreak is gaining momentum if growth patterns are shifting from linear to exponential, and if certain areas are experiencing higher mortality rates compared with others. To inform effective health policies and practices, understanding future developments is often more valuable than assessing past events. In response to this need, we have developed enhanced surveillance metrics that capture the dynamic nature of a pandemic and provide insights into imminent growth. Crucially, these metrics indicate where a particular country stands on the epidemiological outbreak curve. In addition, we incorporate dynamic metrics that measure the pace of the pandemic at the province, territory, and national level. This includes assessing how the acceleration of the pandemic’s speed this week compares with the previous week and how newly reported infections last week can predict new cases in the current week. We can think of this predictive measure as the propagation of cases into the future. These metrics have undergone testing and validation in previous research [5,7,8,17].

For purposes of this study, standard surveillance metrics help us understand past developments in Canada, while enhanced surveillance metrics provide insights into future developments and the position of provinces or territories along the epidemiological curve. Both types of metrics are used to analyze the potential conclusion of the pandemic within Canada. This study used these metrics to assess whether the COVID-19 pandemic in Canada exhibited expansion or contraction around the declaration by the WHO, which marked the conclusion of COVID-19 as a public health emergency of international concern on May 5, 2023. We analyzed changes in the transmission rate to identify any acceleration or deceleration in the pandemic. It is important to note that statistical insignificance is significant in this context; it may indicate the epidemiological “end” of the pandemic if the rate of new cases is negligible and stable, signifying neither acceleration nor deceleration.

Next, we used dynamic and genomic surveillance methods to construct a historical narrative of the pandemic within the country. This analysis incorporated the ratio of COVID-19 deaths to the number of transmissions as an indicator of population-level mortality risk from infection. In addition, we integrated historical genomic surveillance data from sequenced viral specimens to trace the emergence and spread of variants of concern within the country.

Finally, this study aimed to provide historical context for the trajectory of the pandemic in Canada. We addressed several questions, including how provinces and territories responded to the pandemic, how Canada fared in terms of disease burden, and the multifaceted impact of social, economic, and political factors on the course of COVID-19 within the country. This contextual analysis serves as a repository that will inform future disease prevention and mitigation strategies during future pandemics.

### Objective

This study is guided by three primary objectives: (1) to assess the status of the COVID-19 pandemic in Canada around the declaration by the WHO; (2) to construct a historical narrative of the pandemic in the country through dynamic and genomic surveillance methods; and (3) to provide social, economic, and political context for the national trajectory of the pandemic.

## Methods

### Overview

This study conducted an epidemiological assessment with longitudinal, Canadian data from 2 sources: Our World in Data (OWID) and the Global Initiative on Sharing All Influenza Data (GISAID) [18,19].

OWID compiles data on COVID-19 cases and mortality from various sources, including individual websites, statistical reports, and press releases. This study provides updates on traditional surveillance data and dynamic panel estimates from the original study by Post et al [5]. The OWID data for Canada comprised a balanced panel of 13 provinces and territories, running from June 19, 2020, to May 5, 2023. As Canada switched from daily to weekly data reports ahead of the WHO declaration, we used a cubic spline to interpolate daily new cases and deaths if any province or territory had 4 consecutive periods of nonzero new cases interspersed by 6 days of zero new cases.

To identify the appearance and duration of variants of concern in Canada, we also used data on sequenced SARS-CoV-2 variants from GISAID, which is an effective online resource for sharing genetic, clinical, and epidemiological COVID-19 data [19-22]. We used Nextclade nomenclature [23] to collect clade designations from sequences and Pangolin nomenclature for lineage designations of SARS-CoV-2 [24,25]. Metadata for the study period were collected on June 22, 2023. To avoid low frequency or potentially erroneous samples, the dataset was further filtered to exclude months with fewer than 100 available samples, variant groups with fewer than 5 samples in a month, and variant groups representing less than 0.5% of the total samples in a month. The final dataset consisted of 184,386 total samples available on GISAID [19-22].

Traditional surveillance metrics include the rate of new COVID-19 cases per 100,000 population, also called the speed of the pandemic. Enhanced metrics include acceleration, jerk, and 1- and 7-day persistence measures. Acceleration is the change in speed from one unit of time to the next. Acceleration can identify whether speed, or the rate of new cases, is increasing (positive acceleration), decreasing (negative), or stable (zero). “Jerk” is the change in acceleration from one time unit to the next. Large, positive values of jerk can indicate explosive growth in transmission rates. The 1- and 7-day persistence metrics capture the statistical impact of the 1- and 7-day lag of speed on current speed. Thus, 1- and 7-day persistence, respectively, measure how COVID-19 cases echo forward to new cases either 1 or 7 days later. They are derived from an Arellano-Bond dynamic panel data model [26]:



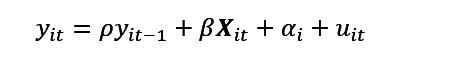



In model 1, speed is the outcome variable. Independent variables include weekend and recent week indicators, while *α_i_* is a fixed effect for province or territory, and *u_it_* is the idiosyncratic error term. For more details, see the original study by Post et al [5].

We analyzed the potential “statistical end” to Canada’s pandemic with a 1-sided *t* test for whether the mean of speed was equal to or greater than the outbreak threshold of 10. We ran the test on a rolling 6-month window over weekly speed for Canada, and we plotted the *P* values from the test over time. All statistical analyses were conducted in R (version 4.2.1; R Foundation for Statistical Computing) with the *plm* package (version 2.6-2) [27,28].

### Ethical Considerations

This study used publicly available data with no identifiable, private information. As defined by the US Department of Health and Human Services Policy for Protection of Human Subjects (section 45CFR46:102), this work, therefore, does not qualify as human subjects research. The Data Availability section provides the data sources.

## Results

Table 1 presents the dynamic panel estimates for the most recent time window. The Wald test for the regression was significant (*P*<.001). Furthermore, the Sargan test did not provide evidence to reject the validity of the overidentification restrictions, with a *P* value close to 1 (*P*≥.99), supporting the reliability of our instrumental variable approach. While the 1-day lag coefficient was statistically significant, suggesting a cluster effect in which cases on a given day impact cases 1 day later, the 7-day lag coefficient was negative and insignificant, suggesting that the broader weekly echo-forward effect of novel COVID-19 cases had largely dissipated by the time of the WHO declaration. The shift parameters for the most recent weeks were indistinguishable from zero, suggesting little change in the status of the pandemic ahead of the declaration. These findings shed light on the evolving nature of the pandemic’s dynamics in the lead-up to May 5, 2023

**Table 1:** Arellano-Bond dynamic panel data estimates for COVID-19 dynamics at the province or territory level in Canada for the week of April 28, 2023.

Variable	Value	*P* value
1-Day persistence coefficient	1.074	<.001
7-Day persistence coefficient	–0.080	.02
Shift parameter week of April 21	0.003	.75
Shift parameter week of April 28	0.018	.31
Weekend	0.001	.88

Static surveillance metrics for the week of April 21, 2023, are provided in Table 2. Similar results for the week of April 28, 2023, are provided in Table S1 in Multimedia Appendix 1. Every province and territory had a small number of new COVID-19 cases. The highest rate of new cases per 100,000 population in either week was 3.67 in Quebec, which is considered a low transmission rate by the Centers for Disease Control and Prevention (CDC) [29]. This rate falls well below the informal outbreak threshold of 10 cases per week per 100,000 population [5,8,30]. Specifically, a “Low” transmission is considered no more than 10 cases per 100,000 people per week. “Moderate” transmission is 10 to 50 cases per 100,000 people per week. “Substantial” transmission is 50 to 100 cases per 100,000 people per week [29,31]. Overall, the status of the pandemic ahead of the WHO declaration in Canada is consistent with an “end” to the pandemic. Based on the definition of a pandemic or an outbreak in several provinces and territories, the data indicate a shift from a pandemic to endemic COVID-19 in Canada.

**Table 2:** Static COVID-19 surveillance metrics for Canadian provinces and territories for the week of April 21, 2023.

Province or territory	New COVID-19 cases, n	Cumulative COVID-19 cases, n	7-Day moving average of new cases	Infection rate per 100,000 individuals	New deaths, n	Cumulative deaths, n	7-Day moving average of deaths	Death rate per 100,000 individuals	Conditional death rate
Alberta	50	631,759	53.43	1.13	2	5701	2	0.05	0.01
British Columbia	91	400,025	83.71	1.77	6	5476	6.86	0.12	0.01
Manitoba	14	155,848	15.57	1.02	1	2493	1	0.07	0.02
New Brunswick	12	90,574	11.43	1.54	1	879	0.57	0.13	0.01
Newfoundland and Labrador	6	55,261	6.29	1.15	0	337	0	0	0.01
Northwest Territories	0	11,511	0	0	0	22	0	0	0
Nova Scotia	23	142,777	25.29	2.35	1	841	0.29	0.10	0.01
Nunavut	0	3,531	0	0	0	7	0	0	0
Ontario	217	1,614,694	226.86	1.47	3	16,488	3.43	0.02	0.01
Prince Edward Island	4	57,226	4.86	2.51	0	100	0.71	0	0
Quebec	315	1,336,972	333.57	3.67	5	17,785	5	0.06	0.01
Saskatchewan	23	155,381	25	1.95	1	1961	0.43	0.08	0.01
Yukon	50	631,759	53.43	1.13	2	5701	2	0.05	0.01

Comparing Table 2 and Table S1 in Multimedia Appendix 1, there was little to no change before and after the WHO declared an end to COVID-19 as a public health emergency of international concern. Without question, Ontario and Quebec had the most cases of COVID-19 transmissions and deaths, but this rank is a function of population size. Thus, a better measure is the number of COVID-19 cases and deaths per 100,000 population. Furthermore, death is often a better proxy for the state of an outbreak than transmissions because deaths are less likely to be undercounted [32]. Undercounting may be due to poor public health infrastructure, home antigen testing, or a dearth of polymerase chain reaction testing or other resources. Ontario and Quebec each reported 0.01 deaths per confirmed case. When we control for the risk of death given the number of COVID-19 transmissions, we find that Manitoba had the highest conditional death rate of 0.02 deaths per confirmed case (rounded to 2 decimal places). The conditional death rate was remarkably consistent across provinces and territories, ranging from approximately 0.00 to 0.02.

Table 3 and Table S2 in Multimedia Appendix 1 contain enhanced dynamic surveillance metrics for the 2 weeks ahead of May 5. Speed was low for every province and territory, and acceleration was almost uniformly either zero or negative. The 7-day persistence effect on speed was also zero or negative. These metrics suggest the pandemic has ended for the country. We note that the figures in Table 3 and Table S2 in Multimedia Appendix 1 are not calculated as day-over-day averages across the week, as they are in Table 2 and Table S1 in Multimedia Appendix 1. Thus, the magnitudes of speed may not exactly match those in Table 2 and Table S1 in Multimedia Appendix 1.

Table 4 compares the 1-day persistence effect on speed for the top five provinces and territories for the weeks of April 21, 2023, and April 28, 2023. In each case, the effect was either zero or negative and close to zero. These metrics support an indication that COVID-19 was well controlled in the country overall.

**Table 3:** Novel surveillance metrics for Canadian provinces and territories for the week of April 21, 2023.

Province or territory	Speed	Acceleration	Jerk	7-Day persistence effect on speed
Alberta	1.21	–0.01	–0.01	–0.09
British Columbia	1.63	0.04	0	–0.11
Manitoba	1.13	–0.05	0.02	–0.13
New Brunswick	1.46	0.02	0.02	–0.13
Newfoundland and Labrador	1.20	–0.05	0	–0.14
Northwest Territories	0	0	0	0
Nova Scotia	2.58	–0.06	–0.01	–0.22
Nunavut	0	0	0	0
Ontario	1.54	–0.04	0.01	–0.15
Prince Edward Island	3.04	–0.27	0.09	–0.49
Quebec	3.89	–0.07	0	–0.34
Saskatchewan	2.12	–0.06	0	–0.19
Yukon	1.21	–0.01	–0.01	–0.09

**Table 4:** The 5 Canadian provinces and territories with the highest 7-day persistence estimate in the weeks of April 21 and April 28, 2023.

Week and province		7-Day persistence
**April 21, 2023**		
	Northwest Territories	0
	Nunavut	0
	Alberta	–0.09
	British Columbia	–0.11
	Manitoba	–0.13
**April 28, 2023**		
	Northwest Territories	0
	Nunavut	0
	Manitoba	–0.09
	Newfoundland and Labrador	–0.09
	Alberta	–0.09

**Figure 1:**
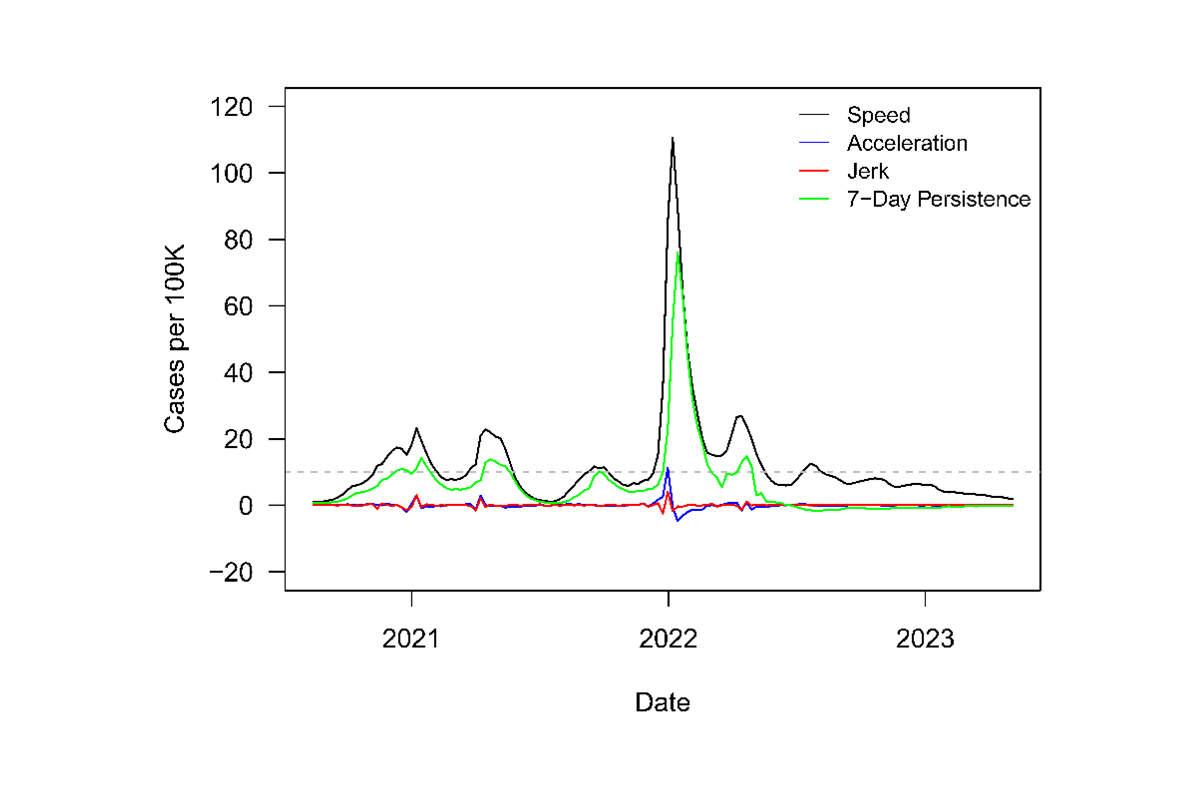
Novel surveillance metrics (speed, acceleration, jerk, and 7-day persistence) for COVID-19 transmissions in Canada from August 2020 to April 2023.

Figure 1 plots national speed, acceleration, jerk, and 7-day persistence metrics from June 19, 2020, to May 5, 2023. The dashed grey line denotes the informal CDC outbreak threshold of speed equal to 10. The country was in and out of outbreaks from November of 2020 until August of 2022. By far, the largest outbreak occurred at the start of 2022. This outbreak, driven by the Omicron variant, brought a peak speed of 110 novel COVID-19 cases per 100,000 population. However, since the outbreak, the country has seen speed tick downward. In fact, Canada had not been in an outbreak for over 8 months before the WHO declaration.

**Figure 2:**
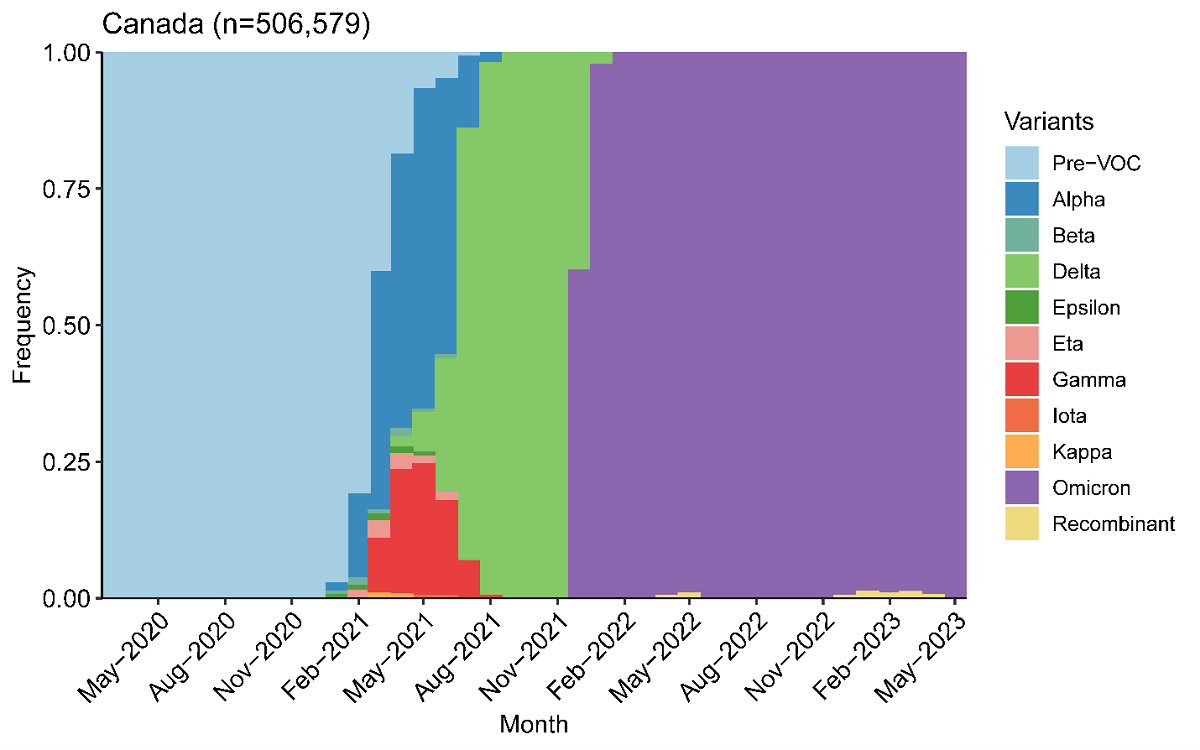
Variants of concern (VOC) as a proportion of all sequenced SARS-CoV-2 specimens from April 2020 to May 2023 in Canada.

Figure 2 plots variant groups as a proportion of all viral specimens collected and sequenced in the country (and made available through GISAID) each month. The country had been in and out of much smaller outbreaks earlier in the pandemic, driven most likely by the ancestral variant, as well as Delta. However, Canada, like much of the rest of the world, saw a surge in cases amid the heightened transmissibility of Omicron [33].

**Figure 3:**
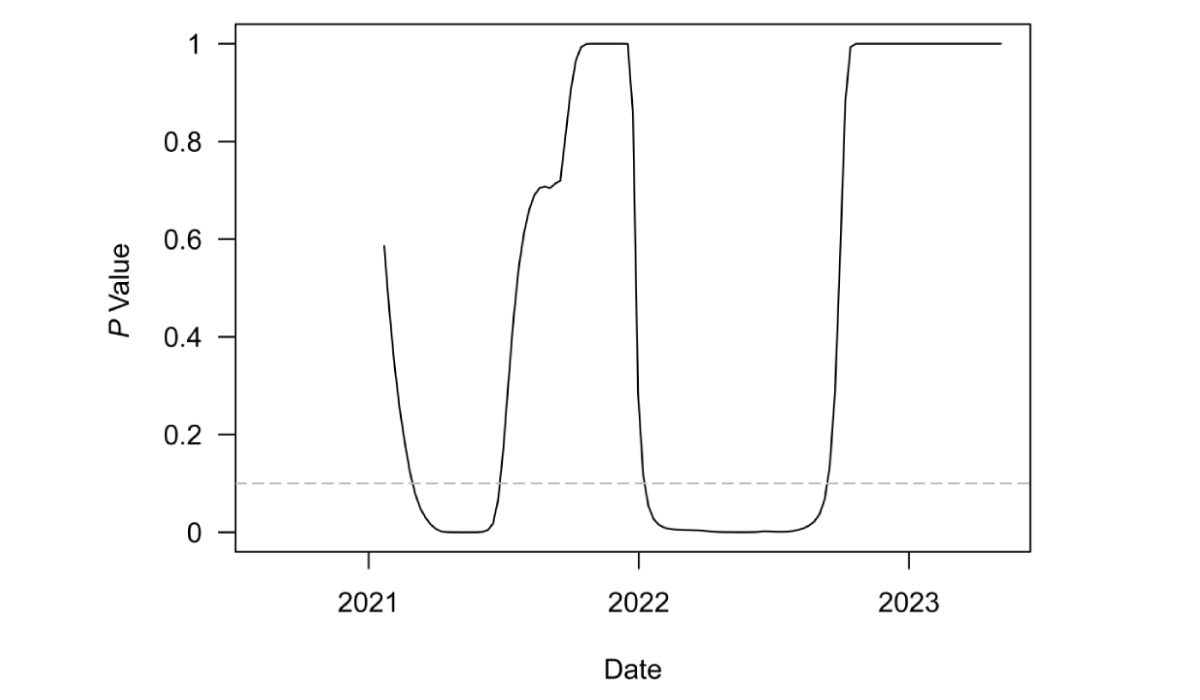
*P* values from 1-sided t tests of weekly COVID-19 transmissions per 100,000 population equal to 10 over a rolling, 6-month window in Canada.

Figure 3 plots *P* values from a series of 1-sided *t* tests of whether Canada’s speed was equal to or greater than the threshold outbreak of 10. These tests were conducted on a rolling 6-month window for weekly national speed. The dashed grey line denotes the least restrictive conventional significance level threshold of α=.10. The test first rejected the null in favor of the alternative for the 6-month period ending in early March of 2021. Soon after, the test became insignificant until it rejected the null again at the start of January 2022. The latter rejection was driven by the Omicron outbreak. The test statistic became totally insignificant from approximately mid-October 2022 onward. This more recent lack of statistical significance is consistent with the end of the pandemic in Canada, as the test clearly failed to reject the null hypothesis of outbreak threshold speed for an extended period.

With the historical context of enhanced surveillance metrics, Canada appears at the end stage of the pandemic. Speed has not been this low for this long since the start of the pandemic.

**Figure 4:**
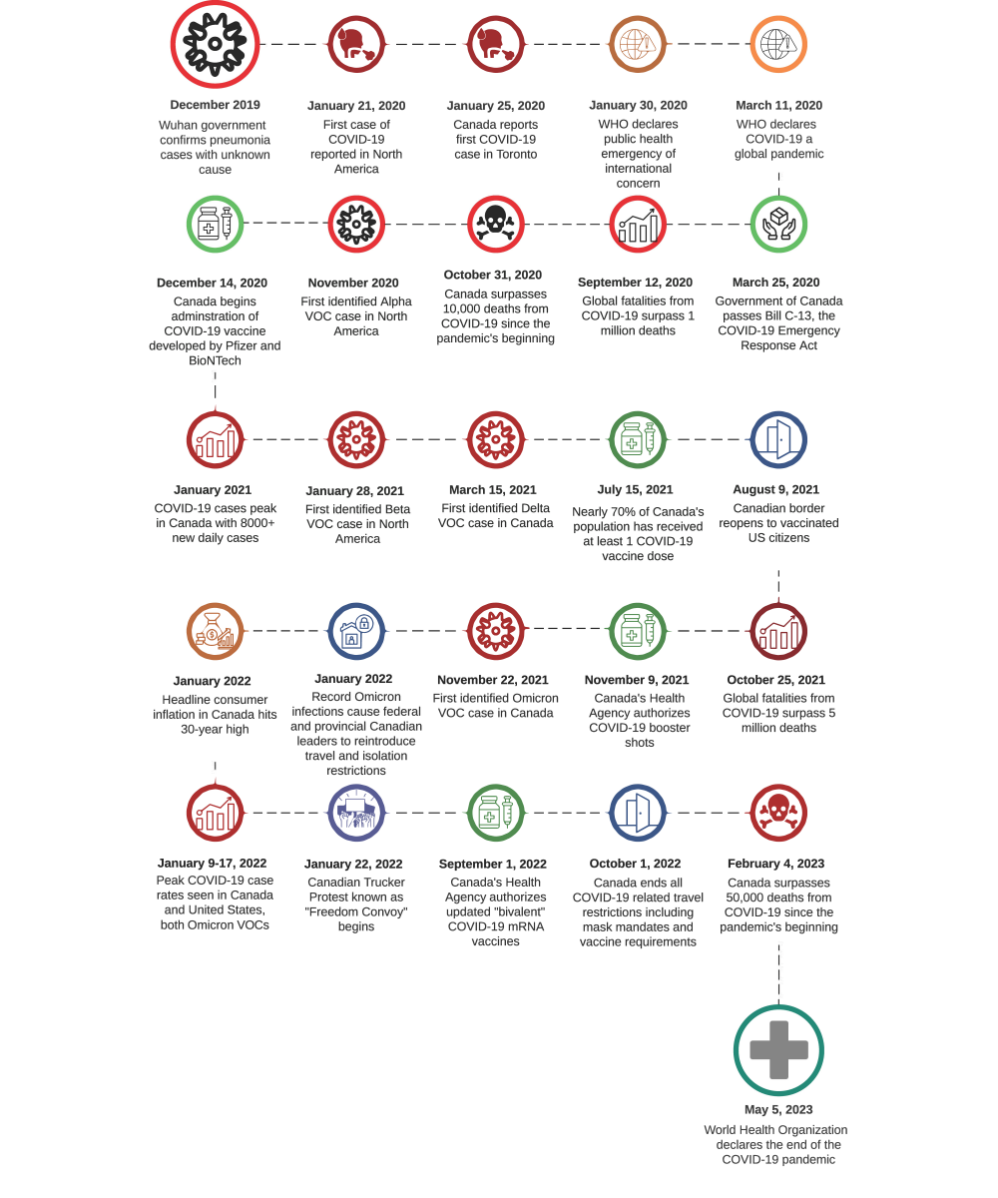
Timeline of the COVID-19 pandemic in Canada. mRNA: messenger RNA; VOC: variant of concern.

Figure 4 provides a timeline of the onset of COVID-19 in Canada, as well as vaccination programs and policy evolution over the course of the pandemic.

## Discussion

### Principal Findings

The first objective of this study was to provide the status of the COVID-19 pandemic in Canada up to the point when the WHO ended its designation of COVID-19 as a public health emergency of international concern. The weekly transmission rate had remained below the outbreak threshold of 10 cases per 100,000 population for approximately 1 year up to the WHO declaration. Furthermore, nearly every sequenced SARS-CoV-2 specimen had been identified as the Omicron variant since January 2021. Dynamic and genomic surveillance methods suggest COVID-19 had transitioned from pandemic to endemic in Canada by the WHO declaration in May 2023.

The first confirmed case of COVID-19 in Canada was reported on January 27, 2020, in Winnipeg [4]. Given Canada’s extensive border with the United States, the timing of outbreaks in both countries closely paralleled each other. However, it is important to note a significant contrast in the scale of these outbreaks. Canada experienced milder impacts than the United States. Canada adopted a range of early pandemic control strategies, with the federal government playing a pivotal role [34,35]. These strategies were characterized by a high degree of unity and stringency, in stark contrast to the fragmented response in the United States [36,37]. As such, early infection and mortality rates in the United States far outpaced those in Canada, with the COVID-19–specific death rate per capita from inception to October 2020 in Canada being just 40% of that in the United States [38,39].

Disease outbreaks during the first wave of COVID-19 in Canada were primarily driven by spread in metropolitan areas such as Montreal [40,41]. Additional outbreaks, caused mainly by new virus variants including Alpha, Beta, Delta, and Omicron, occurred in both urban and rural settings, with subsequent waves of infection occurring during summer 2021, winter 2021, summer 2022, and winter 2022 [8,42,43]. Case volume peaked in January 2022 with the Omicron variant, which was associated with more than 1 million new cases per day in North America at its height [42,43]. Despite formal approval of multiple COVID-19 vaccines by the US Food and Drug Administration and Health Canada, Canada’s Department of National Health Policy responsible for drug and vaccine inspection, there continues to be considerable skepticism regarding vaccine safety and efficacy in North America [44,45].

Sociopolitical conflict accompanied the COVID-19 pandemic in Canada. Despite perceived agreement and compliance with public health recommendations, various social protests have emerged, including a group of shipping truckers known as “The Freedom Convoy 2022,” protesting Canada’s federal vaccine mandate by disrupting shipping flow in Ottawa [46]. This protest resulted in Prime Minister Justin Trudeau invoking the country’s Emergencies Act for the first time since its ratification in 1988 [47]. The statute, which allows Canada’s federal government to take extraordinary measures in responding to public welfare emergencies, was used to restrict travel to Canada and to disband the convoy’s protests [47].

In addition, COVID-19 brought significant financial consequences to Canada [48]. Key financial measures taken included the Bank of Canada reducing interest rates and extending bond buyback programs, as well as the federal government redirecting CAD $290 billion (13.2% of Gross Domestic Product, approximately US $209 billion using exchange rates on November 11, 2024) to households and companies in the form of wage subsidies, tax credits, and tax deferrals [48]. While Canada was relatively successful in mitigating economic disruption, it was among the top international fund borrowers from the International Monetary Fund’s Group of 10 financial assembly [49].

### Policies Implemented to Control and Mitigate the Transmission of COVID-19

Canada implemented numerous initiatives to mitigate COVID-19 infections and reduce mortality rates. The country’s provision of publicly funded health services facilitated an early, swift, and robust response to the pandemic [50]. Responses included optimizing hospital capacity, implementing intensive care training for various clinicians, and constructing new testing and screening facilities [50]. The country’s response was largely informed by its previous experiences with the 2003 SARS pandemic, which was credited with highlighting various previous vulnerabilities in Canada’s health care system [34]. Canada was also praised for its stringent border control measures and subsequent border vaccination requirements, as well as its strategic school, transport, and public event closures [49]. Due to the shared border and frequent international travel, Canadian success in disease control and mitigation was somewhat tempered by the relative lack of success in the United States [51].

### Limitations

COVID-19 data had become less frequently reported around the world by the time the WHO declared an end to COVID-19 as a public health emergency [52]. Canada switched to weekly instead of daily data reports, which introduces uncertainty in the daily estimates generated from cubic spline interpolation. In addition, more people began to use at-home tests as the pandemic evolved [53]. Viral specimen tests for variants of concern in GISAID are also dependent on testing and sequencing capacity, which varied by province and territory across the country.

### Conclusions

The concern about potential resurgences of the virus remains valid. As long as COVID-19 continues to spread and mutate, the possibility of new variants emerging remains. Variants could potentially be more transmissible, resistant to vaccines, or cause more severe illness. This underlines the importance of continued vigilance, vaccination efforts, and global cooperation to control the spread of the virus [54].

Ahead of eventual future pandemics, one of the more important takeaways from the COVID-19 pandemic is how a nation can reduce disease burden before vaccines and treatment modalities become widely available [55]. Canada was relatively proactive in border control measures and lockdowns compared with its neighbor, the United States, and these measures were effective [5,6,49]. Other research indicates that rapid testing of individuals led by an epidemiological task force should be another first line of defense [56]. New technologies for surveillance, such as wastewater management, may offer ways to accelerate early outbreak detection [57-59]. Finally, because of shared borders and increased international travel, continued cooperation among nations will be needed to reduce the likelihood and disease burden from pandemics in the future [60,61].

## Appendix

Multimedia Appendix 1 Static and novel COVID-19 surveillance metrics for Canadian provinces and territories for the week of April 28, 2023.

## Abbreviations

CDC: Centers for Disease Control and Prevention
GISAID: Global Initiative on Sharing All Influenza Data
OWID: Our World in Data
WHO: World Health Organization

## Data Availability

The datasets analyzed during this study are available in the Our World in Data repository [18] and the Global Initiative on Sharing All Influenza Data (GISAID) [19].
