# Supplementary material for: Updated Surveillance Metrics and History of the COVID-19 Pandemic (2020-2023) in Canada: Longitudinal Trend Analysis
Source: JMIR Public Health Surveill. 2024 Dec 5;10:e53218. doi: 10.2196/53218 (PMC11659694; doi:10.2196/53218)
Supplement: Multimedia Appendix 1 [file publichealth_v10i1e53218_app1.docx]

**Multimedia Appendix 1**

**Table S1. Static COVID-19 surveillance metrics for Canadian provinces/territories for the week of Apr 28, 2023.**

| Province/Territory | New COVID-19 cases, n | Cumulative COVID-19 cases, n | 7-day moving average of new cases | Infection rate per 100K individuals | New deaths, n | Cumulative deaths, n | 7-day moving average of deaths | Death rate per 100K individuals | Conditional death rate |
| --- | --- | --- | --- | --- | --- | --- | --- | --- | --- |
| Alberta | 45 | 632,100 | 46.43 | 1.02 | 1 | 5,717 | 1.57 | 0.02 | 0.01 |
| British Columbia | 71 | 400,660 | 82.57 | 1.38 | 5 | 5,520 | 5.29 | 0.10 | 0.01 |
| Manitoba | 15 | 155,944 | 14.29 | 1.09 | 0 | 2,499 | 0.57 | 0 | 0.02 |
| New Brunswick | 1 | 90,652 | 6.43 | 0.13 | 0 | 883 | 0.71 | 0 | 0.01 |
| Newfoundland and Labrador | 8 | 55,305 | 7 | 1.53 | 0 | 337 | 0 | 0 | 0.01 |
| Northwest Territories | 0 | 11,511 | 0 | 0 | 0 | 22 | 0 | 0 | 0 |
| Nova Scotia | 24 | 142,941 | 23.14 | 2.45 | 1 | 850 | 1 | 0.10 | 0.01 |
| Nunavut | 0 | 3,531 | 0 | 0 | 0 | 7 | 0 | 0 | 0 |
| Ontario | 207 | 1,616,208 | 211.71 | 1.40 | 4 | 16,511 | 3.43 | 0.03 | 0.01 |
| Prince Edward Island | 0 | 57,251 | 2.29 | 0 | 0 | 103 | 0 | 0 | 0 |
| Quebec | 284 | 1,339,145 | 297 | 3.31 | 4 | 17,817 | 4.71 | 0.05 | 0.01 |
| Saskatchewan | 20 | 155,543 | 21.71 | 1.70 | 1 | 1,965 | 1 | 0.08 | 0.01 |
| Yukon | 45 | 632,100 | 46.43 | 1.02 | 1 | 5,717 | 1.57 | 0.02 | 0.01 |

**Table S2. Novel surveillance metrics for Canadian provinces/territories for the week of Apr 28, 2023.**

| Province/Territory | Speed | Acceleration | Jerk | 7-day persistence effect on speed |
| --- | --- | --- | --- | --- |
| Alberta | 1.05 | -0.02 | 0.01 | -0.09 |
| British Columbia | 1.60 | -0.06 | -0.02 | -0.13 |
| Manitoba | 1.04 | 0.01 | 0 | -0.09 |
| New Brunswick | 0.82 | -0.20 | -0.04 | -0.11 |
| Newfoundland and Labrador | 1.34 | 0.05 | 0 | -0.09 |
| Northwest Territories | 0 | 0 | 0 | 0 |
| Nova Scotia | 2.36 | 0.01 | 0.03 | -0.20 |
| Nunavut | 0 | 0 | 0 | 0 |
| Ontario | 1.44 | -0.01 | 0 | -0.12 |
| Prince Edward Island | 1.43 | -0.36 | -0.09 | -0.23 |
| Quebec | 3.46 | -0.05 | 0 | -0.30 |
| Saskatchewan | 1.84 | -0.04 | 0 | -0.16 |
| Yukon | 1.05 | -0.02 | 0.01 | -0.09 |
